# Polyphenols from *Inula oculus-christi* L. Induced Cell-Specific Membrane and Cytoskeleton Reorganization

**DOI:** 10.3390/membranes15120357

**Published:** 2025-11-26

**Authors:** Ralitsa Veleva, Aneliya Kostadinova, Antoaneta Trendafilova, Viktoria Ivanova, Veselina Moskova-Doumanova, Kirilka Mladenova, Jordan Doumanov, Dayana Benkova, Galya Staneva, Tanya Topouzova-Hristova

**Affiliations:** 1 Faculty of Biology, Sofia University ‘St. Kliment Ohridski’, 8 Dragan Tzankov Blvd., 1164 Sofia, Bulgaria; ralitsa_veleva@biofac.uni-sofia.bg (R.V.); moskova@biofac.uni-sofia.bg (V.M.-D.); k_mladenova@biofac.uni-sofia.bg (K.M.); doumanov@biofac.uni-sofia.bg (J.D.); 2 Centre of Competence “Sustainable Utilization of Bio-resources and Waste of Medicinal and Aromatic Plants for Innovative Bioactive Products” (BIORESOURCES BG), 1000 Sofia, Bulgaria; antoaneta.trendafilova@orgchm.bas.bg; 3 Institute of Biophysics and Biomedical Engineering, Bulgarian Academy of Sciences, Acad. G. Bonchev Str., Bl. 21, 1113 Sofia, Bulgaria; aneliakk@yahoo.com (A.K.); dayanabenkova369@abv.bg (D.B.); gstaneva@bio21.bas.bg (G.S.); 4 Institute of Organic Chemistry with Centre of Phytochemistry, Bulgarian Academy of Sciences, 1113 Sofia, Bulgaria; viktoria.genova@orgchm.bas.bg

**Keywords:** *Inula oculus-christi* L., polyphenols, caffeoylquinic acids, flavonoid glycosydes plasmalaemma

## Abstract

Interrelations between the plasma membrane and cytoskeleton are of crucial importance for essential cellular processes such as endocytosis, formation of intercellular junctions, cell morphology, etc. Many studies validate the beneficial effects of polyphenols as antioxidant and protective agents, but a molecular mechanism of their interaction and transition through the plasma membranes of different cell lines is still missing. In this study, we examined the affinity of fractions enriched in flavonoid glycosides (FGs) and caffeoylquinic acids (CQAs), obtained from the methanol extract of the medicinal plant *Inula oculus-christi* L., to reorganize the plasma membrane structure and actin cytoskeleton by using confocal microscopy. Assessment of the degree of membrane ordering aiming to distinguish the ordered from disordered regions of the cellular membranes was performed using the fluorescent dye Di-4-ANEPPDHQ, and visualization of F-actin was by TRITC-phalloidin. Two epithelial cell lines with clear differences in their origin and plasma membrane organization were chosen: the non-malignant MDCK II and the cancerous A549. Our results showed that flavonoid glycosides exhibited an ordering effect on plasma membranes of cancerous cells and fluidized one on non-malignant cells. Different patterns of actin reorganization were observed for both cell lines after treatment. Our results indicate the potential of plant-derived polyphenols as modulators of the membrane’s structural organization, offering valuable insights for the development of membrane-targeted therapeutic strategies.

## 1. Introduction

Plants of the genus *Inula* have long been utilized in ethnomedicine across Asia, Europe, and North America due to their diverse therapeutic benefits [1]. Notably, *Inula* sp. plants possess pronounced anti-inflammatory activity which has led to their widespread empirical application as expectorants and antitussives in the treatment of respiratory ailments, including lung inflammation, asthma, cough, sputum production, and wheezing [2].

The health-promoting properties of *Inula* plants are largely attributed to their high phenolic content. Of particular phytopharmacological interest are phenolic acids (PAs) and flavonoid glycosides (FGs). Both compound classes exhibit well-documented biological activities, including antioxidant [3,4,5,6,7,8], anti-inflammatory, and anticancer effects [9,10,11,12,13]. The cell-protective effects of these polyphenols have been partially attributed to their interactions with cellular compartments, thereby modulating key cellular processes. In particular, the cell membrane represents a primary target for the molecular therapeutic actions of various plant-derived polyphenols.

The eukaryotic cellular membrane is composed of a phospholipid bilayer with embedded proteins, creating a heterogeneous landscape with important structural and functional roles [14]. Specific molecular and cooperative lipid–lipid and lipid–protein interactions give rise to lateral segregation and formation of specialized and highly ordered membrane domain “rafts” in a Lo-like (liquid-ordered) phase state enriched in cholesterol, sphingomyelin, and raft-associated proteins. These domains co-exist with a milieu of fluid and disordered Ld-like (liquid-disordered) phases composed predominately of saturated phospholipids [15,16,17,18]. Structurally, membrane rafts range from nanoscale domains that can cluster upon extracellular stimulation to larger assemblies spanning hundreds of nanometers to several microns in size [17,19]. Functional raft macrodomains play crucial roles in diverse cellular processes, including signal transduction, protein sorting, membrane trafficking, and cell adhesion [20,21,22,23].

The implementation of these vital cellular processes requires synergetic interaction between membrane rafts and the cell cytoskeleton. Cortical actin filaments criss-cross with the cytoplasmic membrane surface, forming a dynamic mesh-like structure. These interactions drive the compartmentalization of the entire plasma membrane (∼100 nm and in the range of 30–230 nm depending on the cell type), within which nanoscale lipid rafts (∼2–200 nm) facilitate the organization and temporal regulation of membrane proteins [17]. Membrane rafts are particularly enriched with cytoskeletal proteins, modulators of the cortical actin cytoskeleton, and with factors that anchor actin filaments to the plasma membrane [24]. Activation signals induce enrichment of actin filaments in the cortical cytoskeleton, which anchor to membrane rafts to modulate the diffusion of adjacent proteins and lipids, increasing molecular order and driving the clustering of initially dispersed nanoclusters into functional platforms [17,24,25,26]. Thus, the interplay between membrane rafts and the cytoskeleton governs both raft assembly and cytoskeletal dynamics, with actin-driven raft clusters exerting multifaceted effects on cellular responses. These include modulation of raft-associated signal transduction pathways, facilitation of extracellular-to-intracellular communication via cytoskeletal components, establishment of connections with the extracellular matrix (ECM), generation of cell membrane polarity, and formation of cell junctions [26,27,28]. Disruptions in this synergism—or alterations induced by bioactive compounds—can trigger remodeling of the cytoskeletal architecture, thereby influencing cell migration, morphology, mechanotransduction, and cell proliferation [24,26].

Given the remarkable therapeutic potential of both phenolic acids and flavonoid glycosides, continued investigation of their effects on membrane dynamics and lipid rafts is essential. Previous studies have examined their interactions with the membrane bilayer, including biophysical and biochemical properties and their modulatory effects on raft formation [29,30,31,32,33,34]. However, the literature remains limited regarding their influence on raft–actin cytoskeleton synergism, despite its critical role in maintaining cell viability and function.

In our previous study, we observed selective cytotoxicity of extracts from the Bulgarian plant *Inula oculus-christi* and examined two fractions enriched in flavonoid glycosides (FGs) and phenolic acids (PAs) derived from the methanol extract on membrane properties using model membranes [35]. We observed specific molecular effects, including alterations in lipid order, fluidity, polarity, and raft domain dynamics. In the present study, we aim to extend our investigation to their effects on raft–actin cytoskeleton interactions in cells. We focus on two epithelial cell lines (one cancerous and one non-cancerous) with distinctly different membrane polarity, raft and actin cytoskeleton organization, as well as the capability to form tight junctions in a monolayer. These findings may provide deeper insights into the mechanisms underlying their biological activities and potential anticancer therapeutic applications.

## 2. Materials and Methods

### 2.1. Plant Material and Plant Extracts

Wild-growing *I. oculus-christi* L. plants were collected in full flowering stage in Bulgaria. Dr. Ina Aneva (Institute of Biodiversity and Ecosystem Research, BAS, Sofia, Bulgaria) identified the plant, and a voucher specimen (SOM 1360) has been deposited in the Herbarium of the Institute of Biodiversity and Ecosystem Research, BAS, Sofia, Bulgaria. Air-dried leaves of *I. oculus-christi* L. were successively extracted with chloroform and methanol at room temperature. Crude extracts were obtained after filtration and evaporation of the solvent. Methanol extract was further fractioned by column chromatography on Sephadex LH-20 using methanol as an eluent as described in [36,37]. Fractions enriched in flavonoid glycosides (FFGs) and in caffeoylquinic acids (FCQAs) were used in this study. The presence of chlorogenic and caffeoylquinic acids, or flavonoid glucosides, in the fractions was confirmed by Thin-Layer Chromatography (TLC) comparison with the isolated compounds (proven by NMR) and authentic standards, as previously reported [36].

### 2.2. Cell Cultures

Two mammalian epithelial cell lines with distinct membrane lipid order were used in this study. MDCK II cells, derived from immortalized normal renal epithelium, possess well-defined ordered membrane regions (lipid rafts), are engaged in tight junctions, and a large number of intercellular contacts, making them a good model for studying membrane polarization. In contrast, A549 cells, derived from non-small-cell lung carcinoma, exhibit a more fluid membrane, do not form tight junctions with each other, and do not polarize in a monolayer.

Cells were maintained under standard conditions at 37 °C in a humidified atmosphere containing 5% CO_2_ in Dulbecco’s Modified Eagle Medium (DMEM), supplemented with 10% fetal bovine serum (FBS) and 1% (*v*/*v*) antibiotic-antimycotic solution (penicillin 100 U.mL^−1^, streptomycin 100 μg.mL^−1^ and amphotericin B 0.25 μg·mL^−1^). Cells were cultured in 25 cm^2^ flasks before experiments. For fluorescence imaging, cells were seeded on sterile coverslips in a 24-well plate at an initial density of 2 × 10^5^ cells·mL^− 1^(or 88,106 cells per cm^2^). Cells were treated with 100 or 200 μg·mL^−1^ for 24 or 48 h and then proceeded onto fluorescent analyses as described below.

### 2.3. Evaluation of the Cytotoxicity of FFGs and FCQAs

To assess the cytotoxicity of the extracts used, we applied crystal violet staining and spectrophotometric measurement as previously described in [38]. This approach circumvents potential complications associated with oxidative enzyme-based assays, which may be affected by plant secondary metabolites. The intensity of crystal violet staining depends on the amount of DNA and proteins in the cells, which is proportional to the total number of cells in each sample. Both FFG and FCQA fractions were tested in increasing concentrations—50, 100, 250, and 300 µg·mL^−1^. Cytotoxicity was evaluated at 24 and 48 h post-treatment.

### 2.4. Di-4-ANEPPDHQ Microscopy Measurements

The degree of membrane order was assessed using an optimized protocol with the fluorescent probe Di-4-ANEPPDHQ [39]. This styryl dye, originally developed as a voltage-sensitive probe to detect electrical activity in cells and tissues, also serves as an environmentally sensitive indicator capable of discriminating between lipid phases in both model membranes and intact cells. The probe localizes deep into the membrane and is cholesterol-dependent. Di-4-ANEPPDHQ was purchased from Thermo Fisher Scientific (Sofia, Bulgaria).

Fluorophore was solubilized in dimethyl sulfoxide (DMSO) to a stock concentration of 5 mM and stored at room temperature in the dark. Stock solutions were used for no longer than one month before being replaced with fresh solution. The optimized protocol described in [38] was followed to obtain micrographs of both cell lines. Prior to microscopy experiments, cells were incubated in serum-free medium for 30 min during which fluorophore was added to achieve 5 µM final concentration. Di-4-ANEPPDHQ staining was visualized using a widefield fluorescent Leica TCS SPE microscope (Leica microsystems, Wetzlar, Germany) equipped with a 63× immersion objective and Leica microsystems LAS AF software version 2.2.1. Generalized Polarization (GP) calculations were based on the equation GP = (I_500–580_ − I_620–750_)/(I_500–580_ + I_620–750_), as previously described [39]. Pseudo-colored GP images were merged with mean fluorescence intensity images to preserve structural information. We used the plugin published in Owen et al., 2011 [39], adapted for the CellTool 3.0 software package by Georgi Danovski (http://dnarepair.bas.bg/software/CellTool/ (accessed on 23 June 2023)). The images were thresholders before GP calculation to optimize the signal-to-noise ratio. Photomultiplier tube (PMT) gains were adjusted to avoid saturation in either channel and to ensure comparable intensity across channels. Because membrane packing assessment is a ratiometric measurement between the two channels, PMT gains were kept constant between treated and untreated polyphenol-exposed cells. Under these conditions, the G factor for sensitivity between channels was close to unity. GP values were calculated from raw fluorescence images and averaged from a minimum of 30 cells per experiment. Each experiment was performed at least three times. Values are expressed as mean ± SEM; the *p* value was computed using Student’s *t*-test. Data with *p* < 0.05 are considered significant [38].

### 2.5. Fluorescent Staining of F-Actin

The cells were treated as described above. Cell monolayers were washed with 1× PBS containing 100 µM CaCl_2_ and 1 mM MgCl_2_, followed by fixation with 4% formaldehyde in PBS for 20 min. Cells were then permeabilized with 0.5% Triton X-100 in PBS for 5 min and blocked with 1.5% bovine serum albumin (BSA) in PBS with 0.1% Tritin X-100 for 30 min. After washing with PBS, cells were stained with TRITC-phalloidin in PBS for 1 h to visualize the actin cytoskeleton. The cell nuclei were stained with 5 µg·mL^−1^ DAPI (4′,6-diamidino-2-phenylindole), (excitation: 358 nm; emission: 461 nm) in Fluoromount.

### 2.6. Statistical Analysis

The statistical analysis was performed using OriginPro 9.0. The mean values of each measured parameter at 37 °C were compared using Student’s *t*-test. Statistically significant differences based on the Tukey test linked to the control group and between the two investigated PPs (FGs and CQAs) were determined at *p* < 0.05 (*), and no significant differences were marked as ns.

## 3. Results

### 3.1. Dominant Compounds in the Fractions Enriched in Phenolic Acids (FCQAs) and Flavonoid Glycosydes (FFGs) from Inula oculus-christi L.

**Figure 1:**
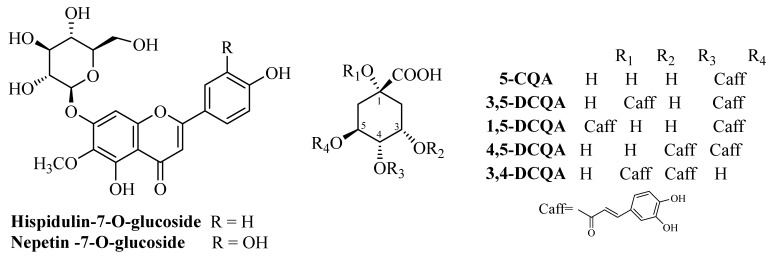
Structures of the dominant compounds in FFGs and FCQAs.

The phytochemical study of the methanol extract of *Inula oculus-christi L.* has already been reported [36,37]. Two fractions were used in this study, namely fractions enriched in phenolic acides (FCQAs) and flavonoid glycosides (FFGs). TLC comparison with previously isolated compounds as standards revealed the presence of chlorogenic (5-caffeoylquinic acid, 5-CQA), 1,5-, 3,5-, 4,5-, and 3,4-dicaffeoylquinic (DCQA) acids in FCQAs and nepitrin (nepetin-7-O-glucoside) and hispidulin-7-O-glucoside (see Figure 1).

### 3.2. Cytotoxicity of the Fractions Enriched in Caffeoylquinic Acids (FCQAs) and Flavonoid Glycosydes (FFGs) from Inula oculus-christi L.

**Figure 2:**
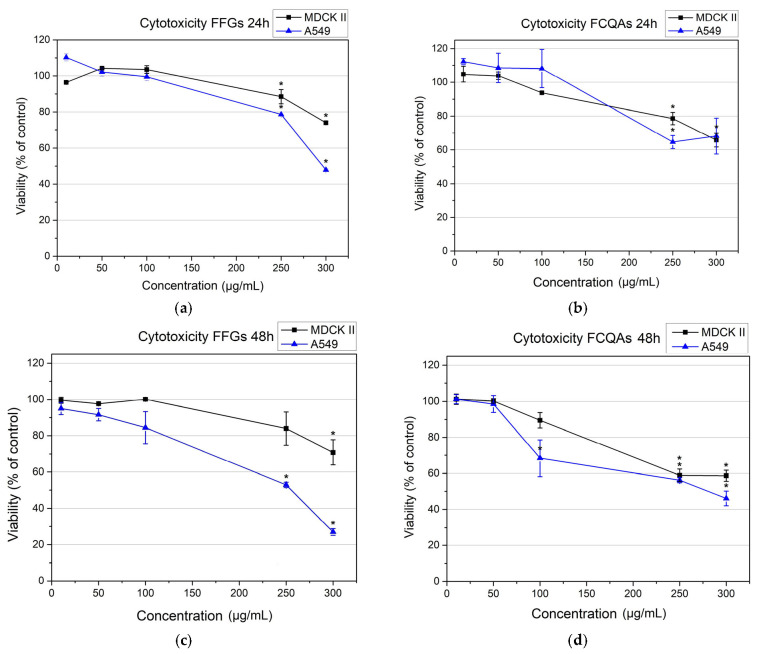
Cytotoxicity of FFGs (**a**,**c**) and FCQAs (**b**,**d**) on MDCK II and A549 cell lines after 24 h (**a**,**b**) and 48 h (**c**,**d**). The data were analyzed with OriginPro 9.0 and presented as a mean value ± SE. Statistical significance (*) is according to one-way ANOVA at the 0.05 level.

Both tested fractions, FFGs and FCQAs, exhibited dose- and time-dependent effects on the cell viability of both cell lines. In the non-cancerous MDCKII cell line, FFG treatment resulted in consistent cell responses at both early (24 h) and late (48 h) time points. At lower concentrations (50–100 µg·mL^−1^), cell viability remained high (~100%), while higher concentrations (250–300 µg·mL^−1^) induced a modest reduction in viability (~75%). No further cytotoxic effects were observed within the tested range, indicating good biocompatibility of FFGs with MDCKII cells (Figure 2). In contrast, FFGs exerted a pronounced dose- and time-dependent cytotoxic effect on the cancerous A549 cell line. At concentrations of 250–300 µg·mL^−1^, a significant decrease in cell viability was detected, with an IC_50_ value of 295 µg·mL^−1^ at 24 h post-treatment. This effect became more substantial over time, with viability dropping to ~50% at 250 µg·mL^−1^ and below 30% at 300 µg·mL^−1^ after 48 h, resulting in a lower IC_50_ value of 256 µg·mL^−1^.

The FCQA fraction demonstrated comparable effects in both MDCKII and A549 cells during early exposure. At lower concentrations (50–100 µg·mL^−1^), minimal to no cytotoxicity was observed. However, at higher concentrations (250–300 µg·mL^−1^), a moderate reduction in viability (65–70%) was noted in both cell lines, indicating moderate cytotoxicity (Figure 2b,d). These results suggest that FCQAs are generally well-tolerated across both cell types within the tested concentration range. At 48 h post-treatment, a clear dose- and time-dependent effect was evident for FCQAs in both cell lines. While MDCKII cells exhibited a slight reduction in viability (maintaining ~60%), a more pronounced cytotoxic response was observed in A549 cells, with an estimated IC_50_ value of 280 µg·mL^−1^ (Figure 2d). Overall, these findings demonstrate that both FFG and FCQA fractions exhibit selective dose- and time-dependent cytotoxicity toward the cancerous A549 cell line, while maintaining favorable compatibility with non-cancerous MDCKII cells.

### 3.3. Membrane Order Imaging of Cells with the Fluorescent Probe Di-4-ANEPPDHQ Through Confocal Microscopy

**Figure 3:**
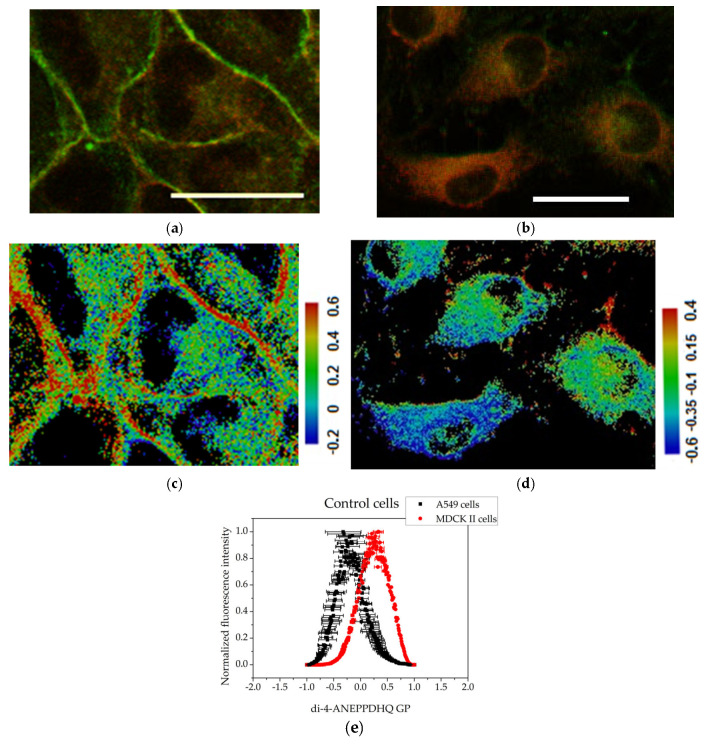
Assessment of the degree of cell membrane order using the fluorescent probe Di-4-ANEPPDHQ excited at 488 nm: (**a**) MDCK II cells merged image of emission at 560 nm (green) and 620 nm (red); (**b**) A549 cells merged image of emission at 560 nm (green) and 620 nm (red). Scale bar—25 µm. GP images of control cells: (**c**) MDCK II cells GP image; (**d**) A549 cells GP image. Scale bar—blue range corresponds to a more fluid lipid state and the red range to the ordered phase, respectively. Summarized data for the generalized polarization of membranes. (**e**) Histograms obtained with the CellTool and Origin programs from GP images of control cells. Mean values of all measurements and standard errors (bars).

**Figure 4:**
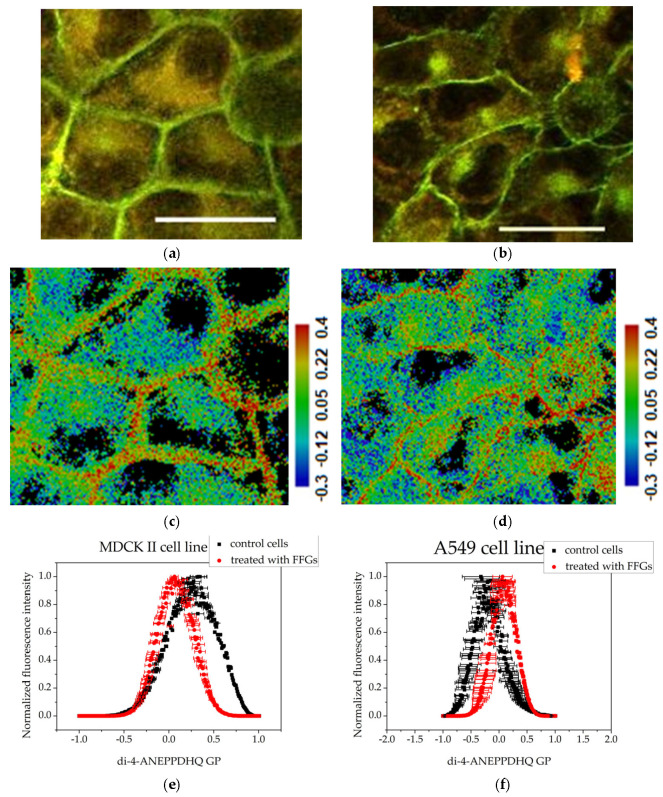
Assessment of the degree of membrane ordering after treatment with 200 μg·mL^−1^ fraction enriched in FFGs using the fluorescent probe Di-4-ANEPPDHQ excited at 488 nm: (**a**) MDCK II cells merged image of emission at 560 nm (green) and 620 nm (red); (**b**) A549 cells—merged image of emission at 560 nm (green) and 620 nm (red). Scale bar—25 µm. (**c**) MDCK II GP image; (**d**) A549 cells GP image. Scale bar—blue range corresponds to more fluid lipid state and the red range to the ordered phase, respectively. Summarized data for the generalized polarization of membranes—(**e**,**f**): histograms obtained with the CellTool and Origin programs from GP images of treated cells. Mean values of all measurements and standard errors (bars).

The membrane lipid order of both MDCKII and A549 cells was visualized using the environment-sensitive fluorescent probe Di-4-ANEPPDHQ. This probe exhibits a fluorescence emission spectrum that is sensitive to the lipid packing and polarity of the membrane environment, properties that are indirectly influenced by the presence of water molecules in more disordered membrane regions. Di-4-ANEPPDHQ partitions evenly between liquid-ordered (L_o_) and liquid-disordered (L_d_) membrane phases but emits from two distinct excited states: a relaxed state associated with loosely packed (disordered) lipids, and a non-relaxed state corresponding to tightly packed (ordered) bilayers. Upon excitation at 488 nm, the probe emits with a peak around 560 nm when located in ordered (L_o_) domains (visualized in green in Figure 3 and Figure 4) and around 620 nm when localized in disordered (L_d_) regions (visualized in red in Figure 3 and Figure 4). This dual-emission behavior enables direct visualization and assessment of membrane organization and lipid order in mammalian cells, including human-derived cell lines [38,39].

In the micrographs of control (untreated) MDCKII cells, a strong green fluorescence signal—corresponding to the liquid-ordered (L_o_) phase—is observed outlining the plasma membrane. As MDCKII cells are polarized epithelial cells, they form well-defined tight junctions and possess raft-enriched plasma membranes. The predominance of the L_o_ phase can be attributed to the high degree of intercellular contacts, which serve as sites for cell–cell adhesion and for anchoring the actin cytoskeleton to the plasma membrane (Figure 3a). The cancerous cell line, A549 (Figure 3b), exhibits a cytoplasm rich in organelles and lamellar bodies, reflecting high metabolic activity and active vesicular trafficking. In contrast to MDCKII cells, the Di-4-ANEPPDHQ fluorescence in A549 cells is predominantly distributed throughout the cytoplasmic membranes, suggesting a lower degree of membrane lipid order at the plasma membrane and possibly higher endocytic or vesicular membrane activity.

Treatment with fractions enriched in FFGs at a concentration of 200 μg·mL^−1^ led to changes in membrane organization in both cell lines. The membranes of MDCK II cells (Figure 4a) retained their distinct line, but noticeable changes in cell shape were observed. The cells appeared more roundish compared to the controls, which is likely a consequence of the loss of some intercellular contacts. Additionally, a more intense fluorescence in the cytoplasm was noticeable. After treatment of the A549 cell line with FFGs (Figure 4b), cells displayed a pronounced fluorescence in the plasma membranes in contrast to the control. The cytoplasm signal from Di-4-ANEPPDHQ was densely concentrated in a specific manner in an area located next to the nucleus.

**Figure 5:**
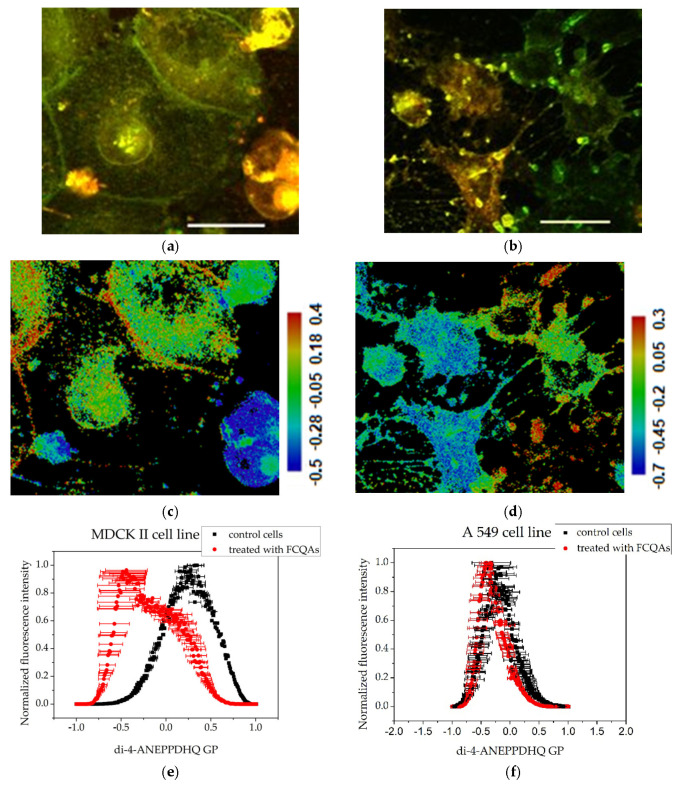
Assessment of the degree of membrane ordering after treatment with 200 μg·mL^−1^ fraction enriched in FCQAs using the fluorescent probe Di-4-ANEPPDHQ excited at 488 nm: (**a**) MDCK II cells merged image of emission at 560 nm (green) and 620 nm (red); (**b**) A549 cells—merged images of emission at 560 nm (green) and 620 nm (red). Scale bar—25 µm. (**c**) MDCK II GP images; (**d**) A549 cells GP images. Scale bar—blue range corresponds to more fluid lipid state and the red range to the ordered phase, respectively. Summarized data for the generalized polarization of membranes—(**e**,**f**): histograms obtained with the CellTool and Origin programs from GP images of treated cells. Mean values of all measurements and standard errors (bars).

Microscopic analysis of MDCKII cells treated with FCQAs revealed notable morphological changes, including a more elongated shape compared to the control group. The cell membranes appeared thinner. In A549 cancer cells, FCQAs caused significant damage. The treated cells exhibited highly altered morphology and extensive vesicle formation, suggesting membrane disintegration (Figure 5).

### 3.4. GP Image Acquisition

The shift in emission spectra of Di-4-ANEPPDHQ can be quantified by Generalized Polarization (GP) analysis. This function (GP) represents normalized fluorescence intensity ratio. The spectral channels corresponding to L_o_ and L_d_ phases are used for the GP equation. Di-4-ANEPPDHQ microscopy measurements and GP calculations are described in Materials and Methods. Quantitative representation of the processed data from the fluorescence micrographs is shown in Figure 3, Figure 4 and Figure 5 and Table 1.

GP images of controls and treated cells are presented in Figure 3, Figure 4 and Figure 5. The blue color range corresponds to a more fluid lipid state and the red color range to a more ordered phase. The generated histograms from the GP images are summarized in Figure 3, Figure 4 and Figure 5. The control MDCK II (Figure 3c,e) cells had higher GP values compared to A549 controls (Figure 3d,e), indicating a more ordered membrane of the MDCK II cells.

Treatment of MDCK II cells with FFG (Figure 4) and FCQA fractions (Figure 5) resulted in increased membrane fluidity compared to control cells (Figure 3), as evidenced by lower GP values. Among the two, FCQAs induced a more pronounced fluidizing effect, as shown in Figure 5 and quantified in Table 1. In contrast, A549 cells exhibited a different response. The FFG had a membrane-ordering effect in these cancer cells (Figure 3), opposite to its effect in non-cancerous MDCK II cells. On the other hand, FCQA treatment led to increased membrane fluidity in A549 cells (Figure 5).

### 3.5. Actin Cytoskeleton Reorganization

**Figure 6:**
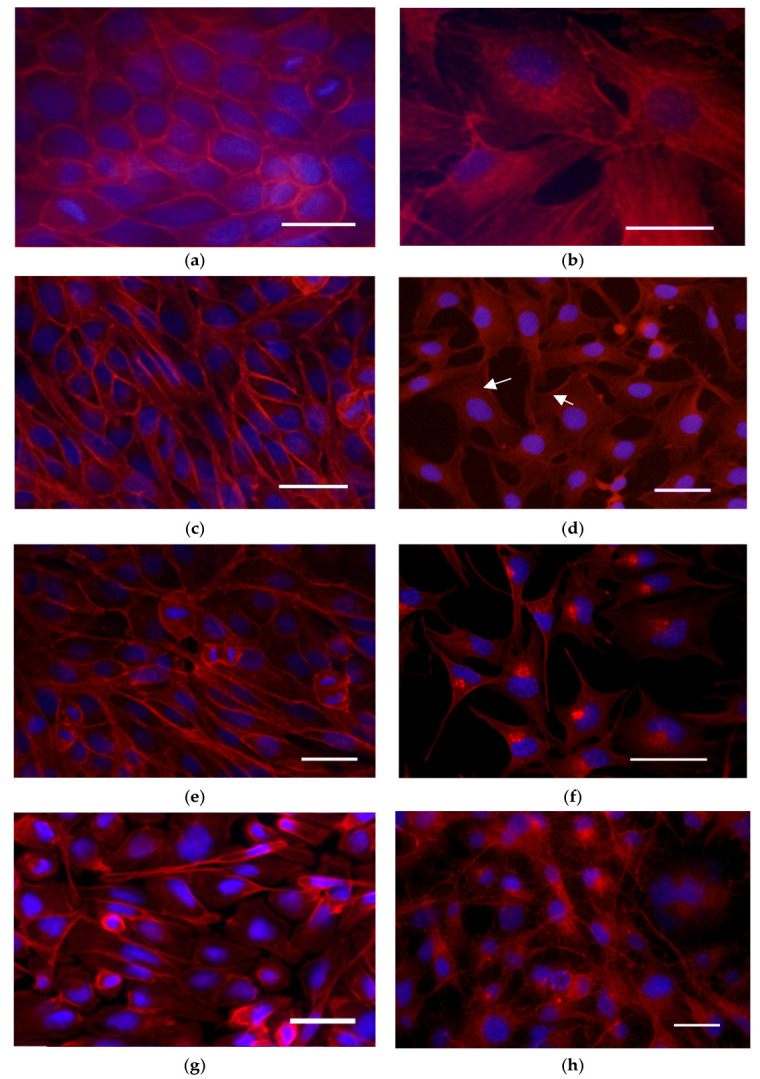
Assessment of the actin cytoskeleton using the TRITC-phalloidine (red) and DAPI (blue): control cells; (**a**) MDCK II cells; (**b**) A549 cells. Cells treated with 100 µg·mL^−1^ FFGs: MDCK II cells at 24 (**c**) and 48 h of treatment (**e**); A549 cells at 24 (**d**) and 48 h (**f**) of treatment. Cells treated with 100 µg·mL^−1^ FCQAs: (**g**) MDCK II cells; (**h**) A549 cells. Staining: TRITC-phalloidine (red) and DAPI (blue). The preparations were observed and imaged using a Nikon TiU confocal laser scanning microscope (Nikon, Tokyo, Japan) and EZC1 software v.3.90. Magnification 400×. Scale bar—25 µm, arrows—actin aggregates.

The cell lines used for this study are epithelial. Fluorescent images of control cells from both cell lines (Figure 6a,b) displayed normal morphology, characteristic features of the respective cell type with confluent monolayer and active cell division. In MDCKII cells (Figure 6a), the fluorescence signal was most intense at the cell periphery and along the plasma membrane. They form many adherent junctions and tight junctions, which ensure the separation of the membrane surface into basolateral and apical. In A549 cells (Figure 6b), a well-structured actin cytoskeleton was observed with pronounced stress fibrils.

Following treatment with FFGs, MDCK cells (Figure 6c,d) continued to exhibit a confluent monolayer with active cell division. Similarly to the control, the fluorescence signal remained most intense in the cell membrane area. In contrast, FFGs reduced the monolayer confluency of the A549 cell line (Figure 6d). After 24 h post-treatment with 100 µg·mL^−1^ FFGs, a redistribution of actin was observed in the cortex of lung carcinoma cells even in areas lacking intercellular contacts. This redistribution persisted after 48 h of treatment (Figure 6e,f). At higher concentrations, the actin rearrangement progressed into distinct cytoplasmic clumping near the nucleus. Comparable clumps were observed in the same cells stained with Di-4-ANEPPDHQ (Figure 4), and GP analysis confirmed that these regions exhibit increased membrane order.

Treatment with fraction enriched in FCQAs resulted in reduced intensity of the signal of F-actin in MDCKII cells (Figure 6g). Actin filaments were generally preserved in the membrane area, where actin aggregates could be found. A549 cells also exhibited decreased fluorescence intensity following FCQA treatment (Figure 6h). These cells were characterized by a branched actin meshwork and the presence of prominent stress fibers.

In general, the tested fractions showed a weaker effect on the actin cytoskeleton of cells from the MDCK II cell line. The A549 line showed great sensitivity to the fractions, as we observed different patterns of disorganization of the actin cytoskeleton upon treatment with the extracts.

## 4. Discussion

The cytotoxicity and biocompatibility of plant-derived polyphenols are largely influenced by their chemical structures and lipophilicity. An increase in the lipophilicity of chlorogenic acids (CGAs) is positively correlated with enhanced cytotoxic effects due to improved membrane permeability and intracellular accumulation [40]. Conversely, naturally occurring CGAs such as 5-caffeoylquinic acid are inherently hydrophilic because of multiple hydroxyl (-OH) and carboxyl (-COOH) groups, which enhance water solubility and contribute to their favorable biocompatibility and selective bioavailability [41]. The presence of a glycosidic moiety at the 7-*O* position imparts amphiphilic properties to the examined flavonoid glycosides [42], which has been shown to correlate exponentially with decreased cytotoxicity, primarily due to limited passive membrane diffusion [43]. In this study, two plant-derived fractions of methanol extracts from Bulgarian *Inula oculus-christi* were used: one enriched in flavonoid glycosides (FFGs) and the other predominantly containing caffeoylquinic acids (FCQAs). The FFG fraction was characterized by flavonoid glycosides such as hispidulin-7-O-glucoside and nepetin-7-O-glucoside, while the FCQA fraction comprised primarily chlorogenic acids (CGAs), including 5-CQA and dicaffeoylquinic acid (diCQA) isomers (1,5-; 3,5-; 4,5-; and 3,4-). According to our results from the cytotoxicity assay, both FFGs and FCQAs fractions demonstrated selective dose- and time-dependent cytotoxicity toward the cancerous A549 cell line, while exhibiting favorable biocompatibility with the non-cancerous MDCK II cells. These findings suggest a degree of cell-type selectivity, which is consistent with previous studies indicating that both chlorogenic acids (CGAs) and flavonoid glycosides (FGs) can exert cell-specific cytotoxic effects towards cancer cells [44,45,46].

At sub-toxic concentrations, both polyphenolic fractions induced alterations in membrane organization and notable rearrangements of the actin cytoskeleton in the examined cell lines. MDCKII membranes exhibited high GP values (+0.331), reflecting enrichment in cholesterol- and sphingomyelin-rich L_o_ domains. This high lipid order aligns with their origin from epithelial cells with many intercellular junctions, including tight junctions [47]. Cortical actin formed a dense mesh beneath the plasma membrane, reinforced by adherent and tight junctions that maintain epithelial integrity. In contrast, A549 cells, derived from lung carcinoma, displayed less ordered, more fluid membranes (GP = −0.323). Elevated membrane fluidity is characteristic of cancer cells and contributes to enhanced motility, invasiveness, and metastatic potential [48]. In these cells, the actin cytoskeleton was organized into prominent stress fibers spanning the cytoplasm, consistent with a migratory phenotype and weaker cell–cell adhesion [49,50]. These clearly distinct differences make both cell lines good models for tracking changes in membrane organization and the actin cytoskeleton in living cells.

The flavonoid glycoside-enriched fraction demonstrated a cell-specific effect on plasmalaema. FFGs exhibited a slightly fluidizing effect on non-cancerous cells (GP = 0.063), but the opposite effect on cancerous cells. However, the membranes of MDCK II cells retained their relatively ordered state without significant alterations in the actin cytoskeleton pattern, and the cellular monolayer was confluent. In contrast, treatment of A549 cells with FFGs caused pronounced actin redistribution in regions lacking intercellular contacts, progressing into highly ordered cytoplasmic clumps near the nucleus. At the same time, the FCQAs induced persistent membrane fluidization in both cell lines: for MDCK II, GP values were −0.441, and in A549 cells we detected minor fluidization (GP = −0.338). The cortical actin filaments in MDCK II cells were mostly preserved, with occasional small actin aggregates near the membrane, while changes in actin organization in A549 lung cells were insignificant.

Our previous findings showed that the same FFGs and FCQAs modulate the phase heterogeneity of model membranes through differential penetration into the lipid bilayer, governed by their chemical structures and degree of lipophilicity [35]. CGAs largely remain at the membrane surface, whereas flavonoid glycosides integrate into the bilayer. These data support the findings of other authors of significant proportions of membrane-incorporated flavonoids compared to those represented in the cytosol when treating colon carcinoma cell line HT-29 with tea flavonoids [51]. The amphipathic FGs embed their hydrophobic aglycone into the membrane core while positioning the polar glycoside near the glycerol backbone [35,52,53], enhancing fluidity in ordered L_o_ domains and increasing order in fluid L_d_ regions [35]. These findings are consistent with our observations on cell membranes. In ordered membranes of non-cancerous MDCK II cells, FFGs could remain near the headgroup region, where they increase local polarity and disrupt lipid packing at this membrane level. In contrast, in more fluid membranes of the cancerous A549 cells, they could penetrate deeper into the hydrophobic core, inducing tighter lipid packing and a subsequent increase in membrane order. On the other hand, hydrophilic CGAs remain primarily in the outer leaflet of the membrane, interacting with lipid headgroups. This increases local polarity at the membrane–water interface and weakens lipid–cholesterol interactions [54,55], as reflected by the elevated GP values observed in both cell lines.

The organization of the actin cytoskeleton, particularly the peripheral cortical actin, is closely linked to the domain organization of the plasma membrane [24,25,26]. Therefore, alterations in membrane order can potentially affect the anchoring and spatial arrangement of actin filaments at the membrane interface. To date, there is limited data on the effects of polyphenols on both membrane order and the actin cytoskeleton simultaneously, with most studies focusing on either membrane properties or actin organization independently [39,56,57].

Lipid raft L_o_ domains are enriched in proteins that tether directly or indirectly to actin filaments, including caveolins, flotillins, integrins, ERM proteins, and GPI-anchored proteins [24,26,27,58]. The tightly packed, ordered lipid environment of these domains enhances the affinity of raft-associated proteins for actin, thereby stabilizing cortical cytoskeleton organization. Disruption or fluidization of the plasma membrane can induce lateral dispersion of lipid raft-associated proteins, weakening their connections to the actin cytoskeleton and leading to actin redistribution or aggregation [24]. A similar phenomenon was observed in MDCK II cells treated with FCQAs. This treatment resulted in a decrease in membrane order, accompanied by partial actin aggregation near the membrane, likely due to disrupted affinity between raft-associated proteins and the actin cytoskeleton. The incorporation of amphiphilic FFGs into the lipid bilayer stiffens the membrane and generates mechanical load, which can be sensed by mechanosensitive complexes, such as integrin-based focal adhesions and cytoskeletal linkers (talin, vinculin, α-actinin) [27,59]. These complexes transmit mechanical cues to the actin cytoskeleton, activating Rho GTPases (RhoA, Rac1, Cdc42), which regulate actin polymerization [60]. The clustering of actin filaments near the nucleus likely represents a mechanosensitive compensatory response to maintain intracellular tension under increased membrane order.

These findings highlight how the initial membrane order, raft composition, and compound chemistry collectively shape lipid raft dynamics and actin organization. They underscore the unique ability of polyphenols to modulate membrane properties, revealing a direct link between membrane order and cytoskeletal architecture that governs cellular responses.

## 5. Conclusions

This study demonstrates distinct effects on membrane organization and cytoskeletal architecture of two polyphenol fractions of methanolic extracts derived from Bulgarian *Inula oculus-christi*—enriched in flavonoid glycosides (FFGs) and caffeoylquinic acids (FCQAs) in both cancerous (A549) and non-cancerous (MDCK II) cell lines. FFGs exhibited selective cytotoxicity toward cancer cells while maintaining biocompatibility with healthy epithelial cells, suggesting potential for therapeutic application.

At a sub-toxic concentration (200 μg·mL^−1^), both polyphenolic fractions induced notable alterations in cell membrane order and actin cytoskeleton dynamics. Prominent effects were observed in the cancerous A549 cell line, where FFGs caused membrane ordering, which was associated with the formation of perinuclear actin aggregates.

The distinct effects of both fractions on membrane–cytoskeleton dynamics across different cell types are likely driven by their specific chemical structures, which determine their mode of interaction with the lipid bilayer. The more hydrophilic nature of FCQAs favors their interaction with the membrane surface of MDCK II cells, where they likely disrupt lipid–lipid and lipid–protein interactions. This leads to increased membrane polarity and a reduction in membrane order. In contrast, the amphipathic properties of FFGs enable deeper insertion into the more disordered plasma membranes of A549 cancer cells, potentially imposing mechanical stress on the cell membrane.

The findings in this study highlight the intricate interplay between membrane biophysical properties, lipid raft–actin cytoskeleton dynamics, and the chemical nature of exogenous compounds. They underscore the potential of plant-derived polyphenols as modulators of membrane-associated signaling and structural organization, offering valuable insights for the development of membrane-targeted therapeutic strategies.

## Figures and Tables

**Table 1:** Peak of histograms obtained from GP images of control and treated cells from MDCK II and A549 cell lines.

Cells	MDCK II—GP Peak at	A549—GP Peak at
Control	0.331 (SE 0.09026)	−0.323 (SE 0.33225)
Treated with FFGs	0.063 (SE 0.04053)	0.047 (SE 0.16069)
Treated with FCQAs	−0.441 (SE 0.23289)	−0.338 (SE 0.07277)

## Data Availability

Data are contained within the article.

## Abbreviations

The following abbreviations are used in this manuscript:

diCQA	Dicaffeoylquinic acid isomers
PA	Phenolic acids
FCQAs	Fraction of caffeoylquinic acids
CGAs	Chlorogenic acids
FFGs	Fraction enriched in flavonoid glycosides
GP	Generalized polarization
